# Impact of Job Engagement on the Quality of Nursing Services: The Effect of Person-Centered Nursing in South Korean Nurses

**DOI:** 10.3390/healthcare9070826

**Published:** 2021-06-29

**Authors:** Hyesun Kim, Kawoun Seo

**Affiliations:** 1 Department of Nursing, Chungbuk National University Hospital, Chungbuk 28644, Korea; twins815@hanmail.net; 2 Department of Nursing, Joongbu University, Chungnam 32713, Korea

**Keywords:** nurses, work engagement, nursing services, patient-centered care, quality of care

## Abstract

Nurses’ job engagement could help improve the quality of nursing services, and person-centered nursing is expected to play an important role in this relationship. However, little is known about the role of person-centered nursing in the association between job engagement and quality of nursing services. This study examines the moderating and mediating effects of person-centered nursing on the relationship between the job engagement and the quality of nursing services in Korean nurses. In October 2020, 200 hospital nurses were surveyed at three university hospitals. The moderating and mediating effects of person-centered nursing were determined using hierarchical regression analysis. There was a significant positive correlation between job engagement, person-centered nursing, and quality of nursing services. Person-centered nursing was found to have a mediating and moderating role in the relationship between job engagement and quality of nursing service. In conclusion, in the impact of job engagement on the quality of nursing service, it plays a buffering role, and the job engagement of nurses improves the quality of nursing services through improvement of person-centered nursing. Therefore, this study recommends the development and implementation of an educational program to foster person-centered nursing in order to improve the quality of nursing services.

## 1. Introduction

Improving the quality of care has always been a focus of Korean hospitals as a means of differentiation in a competitive landscape [1]. The COVID-19 pandemic has brought increased scrutiny causing leaders of medical institutions to focus considerable effort on improving the quality of medical care and the management of medical professionals [2]. Nurses, who account for the largest proportion of specialized medical professionals in hospitals, interact with patients around the clock while providing nursing services [3]. Since nursing service delivery requires spending considerable time with the patient, the nurses have a significant influence on patients’ safety and satisfaction [4]; therefore, efforts to improve nursing service quality are needed. The quality of nursing services refers to the level of a series of nursing practices performed by nurses for patients [5]. For example, the level of patient safety nursing is also a criteria for evaluating the quality of nursing services [4]. In a previous study, the efficiency and productivity of hospital organizations were primarily emphasized as factors affecting this quality [6].

Recently, an important factor influencing the quality of nursing services has emerged—job engagement—which represents an individual aspect of nurses’ work [4]. Job engagement entails a positive and fulfilling mindset and is characterized by vigor, dedication, and absorption in one’s work [7]. In order to improve the quality of nursing services, it is important to maximize the competence and expertise of nurses through motivation [8]. In this context, job engagement can improve the quality of nursing services by motivating nurses to maximize competence and expertise [4,9]. Prior studies have shown engagement levels to be indicative of positive outcomes. Nurses that experienced high levels of job engagement were shown to show an increase in caring behavior, job satisfaction and employee productivity [10,11]. Nurse engagement levels have also been associated with burnout and decreased turnover intension [3,12]. Nurse turnover is a major problem in the current nursing industry in Korea, and this increase in turnover leads to loss of professional nursing personnel, which ultimately leads to a decrease in the quality of nursing services [13].

Meanwhile, person-centered care has been adopted in recent years in the context of improving the quality of nursing services in Korea. The World Health Organization and the U.S. National Institute of Medicine have also proposed a person-centered approach as a key factor in improving the quality of health care [14]. It represents a holistic approach to care that recognizes and respects people as unique individuals, and is used interchangeably with the concept of “patient-centered” care [15,16]. In this context, it can be considered that improving nurses’ perceptions of person-centered nursing can improve the quality of nursing services provided to patients [1,17].

Job engagement generates positive outcomes for patients in a safer and more cost-effective way, based on autonomy and trust [18]. Greater job engagement among nurses leads to fewer errors, higher patient safety, and higher patient satisfaction, resulting in higher quality of nursing services [19]. However, since the core of nursing practice is the individual or “person” [20], it may be inappropriate to state that the quality of nursing services can be improved simply by a nurse focusing on their work. Nurses with high job engagement provide nursing services by taking into account their patient’s situation, perspective, and understanding, in order to improve their work, which is expected to lead to improvement in the quality of nursing services received by them [21]. In fact, previous studies have confirmed the relationship between nurses’ job engagement and their caring behavior and emotional intelligence [10,22]. High emotional intelligence is reflected in a nurse’s ability to display empathy and form a trusting relationship with the patient [23]. Therefore, it can be assumed that increased engagement will improve the quality of nursing services through person-centered nursing. It is also conceivable that person-centered nursing may play a mediating role between job engagement and the quality of nursing services, preventing the decline in quality of nursing services, even for nurses with low job engagement. As such, person-centered nursing is expected to play varied roles in the impact of job engagement on the quality of nursing service, but studies that investigate the role of person-centered nursing in the relationship between job engagement and quality of nursing service are insufficient.

Therefore, this study aims to provide crucial quantitative evidence for improving the quality of nursing services, by clarifying the role of person-centered nursing in the relationship between nurses’ job engagement and quality of nursing services in Korea.

## 2. Aims and Scope

The purpose of this study is to verify the moderating and mediating effects of person-centered nursing in a specific relationship between job engagement and quality of nursing services in clinical nursing.

## 3. Materials and Methods

### 3.1. Design and Participants

The study employed a cross-sectional descriptive design with convenience sampling. Participants included Registered Nurses working in patient care at three university hospitals in South Korea. Inclusion criteria were as follows: (a) nurses who primarily cared for patients in a university hospital, (b) nurses who understood the purpose of the study and agreed to voluntarily participate, and (c) nurses with ≥6 months of clinical experience. Exclusion criteria included (a) nurses who did not engage primarily in patient care, (b) nurses with <6 months of clinical experience, and (c) nurses who did not agree to participate in this study. The required sample size was calculated using G*power program 3.1.9.7 (Universität Düsseldorf, Düsseldorf, Germany). For multiple regression analysis, the minimum number of participants required was calculated to be 162, specifying a power of 0.90, significance level of 0.05, medium effect size of 0.15, and 13 predictors. However, data were collected from 200 individuals considering recovery rate and response fidelity.

### 3.2. Procedure

This study was conducted after obtaining approval from the Institutional Review Board of J University, a research institution. The data were collected from 1–30 October 2020, using self-report questionnaires. Before conducting the survey, the researcher visited the department of nursing at the university hospital and explained the necessity and purpose of the study as well as the contents of the questionnaire to the head nurse of the ward. The nurses who agreed to participate in the study, after hearing the research purpose from the head nurse, provided written informed consent by a form, which also communicated the purpose of the research, the contents of data collection, and the condition of confidentiality of individuals. Afterwards, the questionnaire was distributed to the nurses through the head nurse of the ward. Mobile coupons were provided to the nurses who completed the survey.

### 3.3. Measurements

The questionnaire comprises a total of 72 items: 10 questions on the participants’ general characteristics and clinical experience, 17 items for measuring job engagement, 25 items for measuring person-centered nursing care, and 20 items for measuring the quality of nursing services.

#### 3.3.1. Job Engagement

Job engagement was measured using the job engagement scale, which was developed by Schaufeli et al. [6] and translated by Lee [24]. This instrument consists of a total of 17 questions. Each question uses a 5-point Likert scale to measure responses, and the higher the total score, the higher is the job engagement. The reliability at the time of development [6] was Cronbach’α = 0.93, and the reliability of the instrument in this study was Cronbach’α = 0.93.

#### 3.3.2. Person-Centered Nursing

Person-centered nursing was measured using a person-centered nursing measurement tool developed by Lee [25]. This instrument consists of a total of 25 questions, measuring responses on a 5-point Likert scale; the higher the total score, the higher is the level of person-centered nursing. When developed [25], the reliability of the instrument was Cronbach’α = 0.94, and the reliability of the tool in this study was Cronbach’α = 0.95.

#### 3.3.3. Quality of Nursing Service

The quality of nursing services was assessed using a tool based on the SERVQUAL model developed by Parasuraman et al. [26]. It was later modified by Lee [27], thereby adapting it to the reality of nursing in South Korea. This instrument consists of a total of 20 questions, and each question is measured on a 5-point Likert scale. The higher the total score, the higher is the quality of service. In Lee’s study [27], the reliability was Cronbach’α = 0.97, and the reliability of the instrument in this study was Cronbach’α = 0.95.

### 3.4. Data Analysis

The data collected in this study were analyzed using IBM SSPS 24.0. Descriptive statistics were used to summarize the data on the general characteristics of the participants and their responses regarding job engagement, person-centered nursing, and quality of nursing services. The differences in job engagement, person-centered nursing, and quality of nursing services according to general characteristics of the participants were analyzed using t-test or one-way analysis of variance (ANOVA). The correlations among the three constructs were measured using Pearson’s correlation coefficients. To analyze the mediating and moderating effects of person-centered nursing in the relationship between job engagement and the quality of nursing services, we performed hierarchical regression analyses, employing the three-step procedure of Baron and Kenny [28]. Sobel test was performed to verify the significance of the mediating effect size. Prior to the regression analysis, we confirmed whether our data satisfied the basic assumptions of regression, namely, normal distribution of residuals, linearity, homogeneity of variance, and multicollinearity.

## 4. Results

### 4.1. Participants’ Characteristics

The general characteristics of the participants are presented in Table 1. A majority of the nurses (91.5%; *n* = 183) were female, with an average age of 32.76 years, and over half identified as single. Moreover, 64% reported not having a religion, 70.5% of participants had completed university studies and 40.5% were working in the operating unit. The participants were largely general nurses (87.5%), and 36.0% had work experience of over 121 months. While 63.0% of the nurses were working in shifts, 53.0% reported a neutral level of satisfaction with pay.

### 4.2. Differences in Levels of Job Engagement and Person-Centered Nursing and Quality of Nursing Services According to Participants’ General Characteristics

Table 2 presents the results of the analysis of the differences in the levels of job engagement and person-centered nursing and the quality of nursing services according to the general characteristics of the participants. Those who were over the age of 45 years (*p* < 0.001), were married (*p* < 0.001), had a master’s degree or higher (*p* < 0.001), were a charge nurse (*p* = 0.001), had more than 10 years of work experience (*p* < 0.001), had a fixed work schedule (*p* = 0.002), and were satisfied with pay (*p* < 0.001) had higher job engagement. Meanwhile, a higher level of person-centered nursing was observed among those who were married (*p* = 0.042), had a master’s degree or higher (*p* = 0.018), had a fixed work schedule (*p* = 0.003), and were satisfied with pay (*p* = 0.029). Higher quality of nursing services was found among nurses who were over the age of 45 years (*p* = 0.008), were married (*p* = 0.006), had a master’s degree or higher (*p* = 0.005), and had more than 10 years of work experience (*p* = 0.016).

### 4.3. Correlation among Job Engagement, Person-Centered Nursing, and Quality of Nursing Services

As shown in Table 3, job engagement has a significant positive correlation with person-centered nursing (r = 0.514, *p* < 0.001) and quality of nursing services (r = 0.495, *p* < 0.001). Person-centered nursing has a significant positive correlation with quality of nursing services (r = 0.811, *p* < 0.001).

### 4.4. Mediating Effects of Person-Centered Nursing in the Relationship between Job Engagement and Quality of Nursing Services

As a result of testing the assumptions of regression analysis before the mediating effect, the Durbin-Watson statistic was found to be close to 2 at 1.757–2.222, indicating that there was no autocorrelation between the residuals. The variance inflation factor was less than 10 at 1.000–1.358, indicating that there was no multicollinearity. In addition, the result of the residual analysis showed that, with regard to the linearity of the model, normality and equal variance of errors were confirmed.

**Figure 1:**
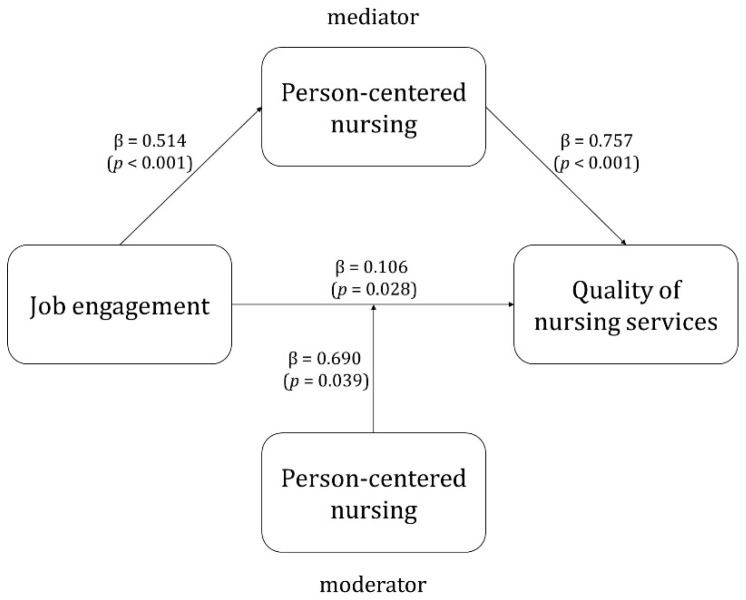
Mediating effects and moderating effects of person-centered nursing between job engagement and quality of nursing services.

The mediating effect of person-centered nursing in the pathway of job engagement affecting the quality of nursing services is shown in Table 4. The result of the first step of the analysis shows that job engagement has a significant influence on person-centered nursing (β = 0.514, *p* < 0.001). The second step of the analysis indicates that job engagement has a significant effect on the quality of nursing services (β = 0.495, *p* < 0.001). In the third step, both job engagement (β = 0.106, *p* = 0.028) and person-centered nursing (β = 0.757, *p* < 0.001) were found to have a significant effect on the quality of nursing services. When person-centered nursing was introduced as a mediator, job engagement still had a significant effect on the quality of nursing services; however, the regression coefficient decreased from 0.26 to 0.06. That is, person-centered nursing partially mediated the relationship between job engagement and the quality of nursing services. Therefore, job engagement had statistically significant direct, indirect, and total effects on the quality of nursing services, while person-centered nursing had a statistically significant direct effect on the latter. The explanatory power of the variable for the quality of nursing service was 66.3%, and it was significant at *p* < 0.001. The Sobel test was performed to verify the significance of the mediating effect of person-centered nursing. This confirmed that it was a partial mediator in the relationship between job engagement and the quality of nursing services (z = 7.460, *p* < 0.001) (see Figure 1).

### 4.5. Moderating Effects of Person-Centered Nursing in the Relationship between Job Engagement and Quality of Nursing Services

The result of the first step of the regression analysis indicates that job engagement has a statistically significant effect on the quality of nursing services (β = 0.495, *p* < 0.001). The second step found that person-centered nursing has a statistically significant effect on the quality of nursing services after adjusting for job engagement (β = 0.757, *p* < 0.001). In the third step, the effect of the interaction between job engagement and person-centered nursing on the quality of nursing service was statistically significant after adjusting for job engagement and person-centered nursing (β = 0.690, *p* = 0.039). The explanatory power of the model for quality of nursing service was found to increase to 24.1% for Step 1, 66.3% for Step 2, and 66.9% for Step 3; that is, person-centered nursing was found to have a moderating effect on the relationship between job engagement and quality of nursing services (Table 5) (see Figure 1).

## 5. Discussion

Efforts to reduce negative psychological factors such as exhaustion and stress of medical staff are continuously being implemented to improve the quality of medical care [10,12,22]. In addition, we are working to enhance positive factors such as job engagement and job satisfaction [10,11,29]. The results of this study showed that there was a significant positive correlation between job engagement and the quality of nursing service. Previous studies of nurses have shown that job engagement is related to nursing job performance and is a factor influencing job performance [30]. For a nurse, job engagement entails forgetting everything other than caring for the patient and focusing solely on the patient [31]. Nurses with high engagement in their role exhibit increased caring behavior as compared to nurses with lower levels of engagement [10]. When nurses focus on their patients it leads to an improvement in the quality of the nursing services they are providing. However, studies of the relationship between job engagement in nursing and the quality of nursing services are still insufficient; further research on this topic should be conducted.

Person-centered nursing was positively correlated with job engagement and quality of nursing services, and it had a mediating effect on the relationship between job engagement and quality of nursing services. This supports our assumption that job engagement improves the quality of nursing services by encouraging the practice of person-centered nursing. According to a previous study, nurses with high emotional intelligence and self-efficacy were found to have high job engagement [22,32]. Emotional Intelligence helps us to understand another person’s point of view and reference [33]. When nurses have a high sense of self efficacy, they are confident and understanding of others, which results in improved nursing services and a higher quality of care [34]. Therefore, nurses with high job engagement can provide a higher level of care. In addition, the quality of care is improved because person-centered nursing leads to the provision of individual level care and a humanistic attitude. Nursing is a physical, mental, emotional, and spiritual act, and hence it is essential that nurses provide person-centered nursing care to effectively perform their duties [35]. This means that the individuals who are the target of nursing care are given more attention and better services, which inevitably leads to a higher quality of nursing services. Thus, the quality of nursing services can be further improved if nurses who experience high levels of engagement in their work adopt a person-centered approach. Therefore, in order to improve the nursing services provided to the patients, job engagement and a person-centered attitude are essential attributes that nurses must possess.

Person-centered nursing has also been shown to have a moderating effect on the relationship between job engagement and quality of nursing services. In other words, person-centered nursing improves the quality of nursing services by interacting with job engagement. Even if they have the same level of job engagement, a nurse with inadequate person-centered nursing attitude may provide low quality of nursing services, and a nurse with a high degree of person-centered nursing attitude may provide high quality of nursing services. This means that, even if nurses are less engaged in their work, adopting a person-centered approach to nursing can act as a buffer and improve the quality of nursing services. These findings can be considered to adequately explain the philosophical underpinnings of nursing [36]. Person-centered care participates in patient decision-making, respects patient choices, and protects patient autonomy and dignity [37]. Therefore, in order to improve the quality of nursing services, it is necessary to improve nurses’ job engagement and awareness of person-centered nursing.

Historically, efforts have been made at the organizational level to manage medical personnel [38]. Recently, many countries have experienced the collapse of their health care system, heightened by COVID-19 [2,39]. As a result, effective leadership of medical personnel, especially by nurse managers has become very important. This study identifies the need for managers to develop more effective human resource management programs to reduce the burnout and turnover of nurses. Successful HRM programs will give nurses more time to collaborate with patients in their care, leading to improved outcomes. Educational programs are another component that can be offered to nurses to improve engagement and knowledge surrounding person-centered nursing.

There are some limitations to consider when interpreting the results of this study. First, it was only conducted among nurses in selected hospitals in South Korea, and since the convenience sampling method was used, care should be taken in generalizing the results. Additionally, this study follows a cross-sectional design and the results will differ depending on how the variables change over time. Therefore, if further studies are conducted on how the variables change after providing interventions for improving person-centered nursing, the causal relationships between the variables can be clearly estimated.

## 6. Conclusions

This study was conducted to clarify the role of person-centered nursing in the relationship between job engagement and the quality of nursing services. There was a significant positive correlation between job engagement, person-centered nursing, and quality of nursing services. Person-centered nursing was found to play a mediating and a moderating role in the relationship between job engagement and quality of nursing services. Based on these results, it is necessary to prepare an educational program to develop person-centered attitudes among nurses. Moreover, in the future, multi-dimensional research on person-centered nursing is needed.

## Figures and Tables

**Table 1:** General characteristics of participant nurses (*N* = 200).

Characteristics	Categories	Mean ± SD or *n* (%)
Gender	Female Male	183 (91.5) 17 (8.5)
Age (years)	≤34	128 (64.0)
	35–44	58 (29.0)
	≥45	14 (7.0)
		32.76 ± 6.61
Marital status	Single	101 (50.5)
	Married	99 (49.5)
Religion	Yes	72 (36.0)
	No	128 (64.0)
Education level	Associate	21(10.5)
	Bachelor	141 (70.5)
	≥Master	38 (19.0)
Work unit	Medical	50 (25.0)
	Surgical	23 (11.5)
	Emergency unit	6 (3.0)
	Intensive care unit	40 (20.0)
	Operating unit	81 (40.5)
Job position	Staff nurse	175 (87.5)
	Charge nurse	25 (12.5)
Work experience (month)	≤60	69 (34.5)
	61–120	59 (29.5)
	≥121	72 (36.0)
		115.18 ± 78.63
Shift type	Fixed	74 (37.0)
	Shift	126 (63.0)
Pay satisfaction	Satisfaction	31 (15.5)
	Neutral	106 (53.0)
	Dissatisfaction	63 (31.5)
Job engagement		4.40 ± 1.01
Person-centered nursing		3.96 ± 0.55
Quality of nursing services		4.06 ± 0.54

**Table 2:** Differences in job engagement, person-centered nursing, and quality of nursing services according to general characteristics of participants (*N* = 200).

Characteristics	Categories	JE	t or F	*P* Tukey	PCN	t or F	*P* Tukey	QNS	t or F	*P* Tukey
Gender	Female	4.38 ± 0.99	−0.64	0.525	3.95 ± 0.56	−0.76	0.451	4.06 ± 0.53	0.30	0.764
	Male	4.55 ± 1.26			4.06 ± 0.47			4.02 ± 0.60		
Age (years)	≤34 ^a^	4.17 ± 0.96	13.60	<0.001	3.91 ± 0.57	2.58	0.079	4.00 ± 0.54	4.93	0.008
	35–44 ^b^	4.66 ± 0.95		c > b > a	3.99 ± 0.50			4.09 ± 0.49		c > a
	≥45 ^c^	5.40 ± 0.97			4.26 ± 0.55			4.45 ± 0.51		
Marital status	Single	4.13 ± 1.00	3.91	<0.001	3.88 ± 0.56	2.05	0.042	3.95 ± 0.55	0.36	0.006
	Married	4.67 ± 0.96			4.04 ± 0.54			4.16 ± 0.50		
Religion	Yes	4.48 ± 1.05	0.87	0.386	3.96 ± 0.53	−0.05	0.958	4.06 ± 0.54	0.89	0.887
	No	4.35 ± 1.00			3.96 ± 0.57			4.05 ± 0.54		
Education level	Associate ^a^	4.36 ± 0.88	16.03	<0.001 c > a,b	3.95 ± 0.36	4.12	0.018 c > b	4.01 ± 0.47	5.42	0.005 c > b
	Bachelor ^b^	4.19 ± 0.96			3.90 ± 0.59			3.99 ± 0.56		
	≥Master ^c^	5.17 ± 0.94			4.19 ± 0.45			4.31 ± 0.43		
Work unit	Medical	4.28 ± 1.06	1.14	0.338	3.84 ± 0.61	2.03	0.091	4.00 ± 0.62	1.75	0.141
	Surgical	4.37 ± 0.90			3.85 ± 0.58			3.88 ± 0.58		
	Emergency unit	4.07 ± 1.24			4.27 ± 0.47			4.44 ± 0.55		
	Intensive care unit	4.25 ± 0.87			3.93 ± 0.50			4.05 ± 0.46		
	Operating unit	4.57 ± 1.06			4.06 ± 0.52			4.11 ± 0.49		
Job position	Staff nurse	4.30 ± 1.00	−3.52	0.001	3.95 ± 0.57	−0.39	0.696	4.03 ± 0.55	0.12	0.114
	Charge nurse	5.05 ± 0.88			4.00 ± 0.40			4.21 ± 0.44		
Work experience (months)	≤60 ^a^	4.13 ± 1.02	9.13	<0.001	3.86 ± 0.59	2.85	0.060	3.96 ± 0.59	4.21	0.016
	61–120 ^b^	4.23 ± 0.91		c > a	3.93 ± 0.55			3.99 ± 0.49		c > a
	≥121 ^c^	4.79 ± 0.98			4.08 ± 0.50			4.20 ± 0.50		
Shift type	Fixed	4.69 ± 1.02	−3.20	0.002	4.11 ± 0.52	−3.02	0.003	4.15 ± 0.52	0.64	0.054
	Shift	4.22 ± 0.97			3.87 ± 0.55			4.00 ± 0.54		
Pay satisfaction	Satisfaction ^a^	4.94 ± 0.76	8.70	<0.001 a > b > c	4.14 ± 0.45	3.61	0.029 a > c	4.21 ± 0.51	4.43	0.607
	Neutral ^b^	4.44 ± 0.83			3.99 ± 0.51			4.10 ± 0.52		
	Dissatisfaction ^c^	4.06 ± 1.27			3.83 ± 0.64			3.90 ± 0.56		

*Note:* JE = Job engagement; PCN = Person-centered nursing; QNS = Quality of nursing services. The differences and order among groups in the post-hoc comparison are indicated by superscript letters (a, b, c).

**Table 3:** Correlation among job engagement, person-centered nursing, and quality of nursing services (*N* = 200).

Variables	1	2	3
1. Job engagement	1		
2. Person-centered nursing	0.514 (<0.001)	1	
3. Quality of nursing services	0.495 (<0.001)	0.811 (<0.001)	1

**Table 4:** Mediating effects of person-centered nursing on the relationship between job engagement and quality of nursing services (*N* = 200).

	β	B	SE	t	*p*	Adj. R^2^	F (*p*)
STEP 1.							
JE → PCN	0.514	0.280	0.033	8.423	<0.001	0.260	70.951 (<0.001)
STEP 2.							
JE → QNS	0.495	0.262	0.033	8.011	<0.001	0.241	64.176 (<0.001)
STEP 3.							
(1) JE → QNS	0.106	0.056	0.025	2.210	0.028	0.663	196.976 (<0.001)
(2) PCN → QNS	0.757	0.736	0.047	15.789	<0.001		

*Note:* Sobel test: Z = 7.460, *p* < 0.001. JE = Job engagement; PCN = Person-centered nursing; QNS = Quality of nursing services.

**Table 5:** Moderating effect of person-centered nursing on the relationship between job engagement and quality of nursing services (*N* = 200).

	β	B	SE	t	*p*	Adj. R^2^	F (*p*)
STEP 1.						0.241	64.176 (<0.001)
JE	0.495	0.262	0.033	8.011	<0.001		
STEP 2.						0.663	196.976 (<0.001)
JE	0.106	0.056	0.025	2.210	0.028		
PCN	0.757	0.736	0.047	15.789	<0.001		
STEP 3.							
JE	−0.392	−0.207	0.129	−1.602	0.111	0.669	134.956 (<0.001)
PCN	0.478	0.465	0.139	3.352	0.001		
JE × PCN	0.690	0.068	0.033	2.075	0.039		

*Note:* JE = Job engagement; PCN = Person-centered nursing.

## Data Availability

The data presented in this study are available on request from the corresponding author. The data are not publicly available due to privacy.
